# An unusual case of presumed cytomegalovirus: retinitis in a non-HIV patient treated for IgG 4-related disease

**DOI:** 10.5935/0004-2749.2022-0230

**Published:** 2025-08-22

**Authors:** Fernando Henrique Flores Teixeira, Alexandre de Carvalho Mendes Paiva, Erika Moreira Carvalho, Nathalia Silva Santos, Ana Luiza Biancardi, André Luiz Land Curi

**Affiliations:** 1 Clinical Research Laboratory of Infectious Diseases, Ophthalmology, Fundação Oswaldo Cruz, Rio de Janeiro, RJ, Brazil

Dear editor,

Immunoglobulin G4-related disease (IgG4-RD) is an immune-mediated, fibroinflammatory condition that can affect any organ system, with a tendency to form tumefactive lesions. It is marked by an elevated serum IgG4^(1,2)^.

Cytomegalovirus (CMV) retinitis is a vision-threatening condition that afflicts immunocompromised patients, especially those with human immunodeficiency virus (HIV) and acquired immunodeficiency syndrome^(3)^. There has not been reports regarding its association with IgG4-RD disease.

Herein, we report a case of a presumed CMV retinitis in a non-HIV patient with IgG4-RD retroperitoneal fibrosis.

A 54-year-old Caucasian woman arrived at the Oswaldo Cruz Foundation Ophthalmology Department, with painless loss of vision in the left eye (OS) for 1 month. She was on treatment for IgG4-RD retroperitoneal fibrosis using 0.9 mg/kg per day of prednisolone for 5 months. Due to a presumptive diagnosis of ocular toxoplasmosis, she was on sulfamethoxazole/trimethoprim (800 mg/160 mg) twice daily for 1 month, without improvement.

**Figure 1:**
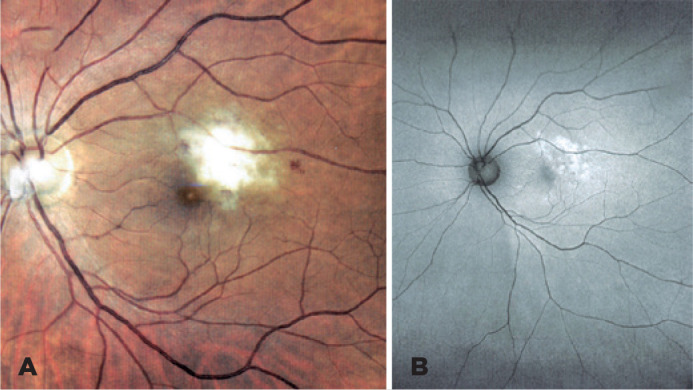
(A) Focal CMV retinitis in the posterior pole of the left eye. (B) Correspondent area of hyperautofluorescence on fundus autofluorescence.

At ophthalmological examination, the best-corrected visual acuity (BCVA) was 20/30 in the right eye (OD) and 20/80 in the left eye (OS). The cornea, anterior chamber, and Goldmann tonometry were unremarkable. Fundoscopy showed no changes in OD, and an area of focal retinitis was found at the posterior pole in OS (Figure 1A).

Previous ancillary tests performed 1 month before were unremarkable in OD. In OS, fundus autofluorescence (FAF) showed a hyperautofluorescent area corresponding to the focal retinitis (Figure 1B), and optical coherence tomography (OCT) showed an involvement of all retinal layers.

Serologic tests revealed positive IgG result for CMV, *Toxoplasma gondii*, syphilis tests (venereal disease research laboratory and fluorescent treponemal antibody absorption test antibodies), HIV, and IgM for CMV were all negative.

**Figure 2:**
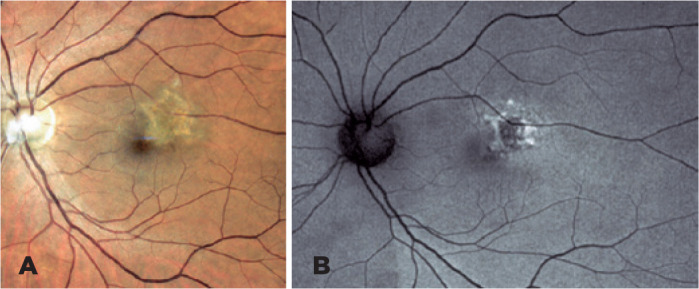
(A) Posttreatment left eye retinography showing CMV retinitis scar. (B) Correspondent area with significant reduction of hyperautofluorescence on fundus autofluorescence.

Methotrexate 25 mg per week and 0.65 mg/kg per day of oral prednisolone were initiated. After 1 month, BCVA improved to 20/50 in OS, and an initial healing of focal retinitis was observed. FAF showed a hypoautofluorescent lesion with hyperautofluorescent borders. After 3 months, methotrexate 25 mg per week and corticosteroid tapering was continued until 5 mg per day, and BCVA improved to 20/40 in OS. The area of focal retinitis was totally healed (Figure 2A), and FAF showed a cicatricial lesion with reduced areas of hyperautofluorescence (Figure 2B).

The hallmark OCT feature due to CMV retinitis is a marked disruption of the retinal architecture, especially at the ellipsoid zone, which is more severe in the area of active retinitis. FAF findings include hyperautofluorescence at the advancing border of the retinitis^(3)^. Both the OCT and FAF examinations performed by the patient matched these findings.

IgG4-RD is characterized by chronic activation of the immune system and tissue fibrosis. The presence of serum autoantibodies and good response to immunosuppression provide support for an autoimmune etiology. Its clinical hallmarks are organ enlargement that mimics a tumor. Glucocorticoids and immunosuppressants are the mainstay of treatment^(1)^.

B-cell polyclonal expansion is a key event in IgG4-RD, which can be proven by the extensive presence of IgG4+ plasma cells in affected tissues. The phenomena is dependent of T-cells, which differentiate B-cells into antibodies producing cells, such as IgG4 antibodies^(1,2)^.

Circulating type 2 helper T-cell (cTh2) is proportional to the number and extension of organ involvement, number of circulating plasmablasts, and serum concentration of IgG4. cTh2 cytokines, such as interleukin (IL)-4 and IL-10 are present in IgG4-RD. Tissues affected with IgG4-RD have shown abundance of T-cells and overexpression of IL-10^(1,2)^.

IL-10 inhibits type 1 helper T-cell (Th1) immune response against viruses^(4)^. CMV produces a homologue IL-10, which helps in evading the immune system and development of retinitis. The homologue CMV IL-10 can bind to the host IL-10 receptor (IL-10R) and exhibit immunosuppressive properties identical to human IL-10^(5)^. Furthermore, receptor haplotypes varied, which result in different susceptibility to CMV retinitis^(5)^.

In the presented case, a presumed CMV retinitis started during IgG4-RD activity. One hypothesis is that elevated serum cTh2 and IL-10 levels lead to the weakening of Th1 cell-mediated immune response, resulting in an active lesion. This fact, associated with possible genetic host factors involving IL-10R, may explain lesion healing and BCVA improvement after immunosuppression with methotrexate.

To our knowledge, this is the first case of a presumed CMV retinitis in a patient with IgG4-RD. This case highlights the importance of the early diagnosis of this condition in patients with IgG4-RD, through physical examination and multimodal assessment.
